# Leech in the Rectum Causing Lower GI Bleeding in a Four Years Old Child: A Case Report

**DOI:** 10.4314/ejhs.v30i6.27

**Published:** 2020-11

**Authors:** Tsion Tilahun, Hawi Babu, Melkamu Berhane

**Affiliations:** 1 Department of Pediatrics and Child Health, Jimma University

**Keywords:** Leech, Bleeding, Hirrudin, Case report

## Abstract

**Background:**

Leeches belong to a group of annelids of the class Hirudinea which are blood feeding ecto-parasites of humans, wild animals and domesticated animals. A leech can suck out as much blood as ten times its own weight. Leech can occur at different sites in humans commonly in the eyes, nasopharynx, larynx, urethra, and vagina and rarely in the rectum.

**Case Details:**

This is a four years old male child who presented with painless, bright red rectal bleeding for two weeks. Heamatocrit was 9.2%. Leech was removed from the rectum by letting the child sit on a bucket of water. The patient was transfused, followed for 24 hours and discharged with iron sulphate syrup.

**Conclusion:**

Leech infestation should be considered in the differential diagnosis of a child presenting with hematochezia.

## Introduction

Leeches make up the third large group of annelids in the class Hirudinea, which are blood feeding ecto-parasites of humans, wild animals and domesticated animals (1). There are over 650 species but only the minority of these are causes of human morbidity (2). Leeches have slender leaf shaped bodies that lack a locomotory organ, bristle like structure called setae (chaetae) and para podia or appendages. Instead of this locomotory organ, the leech possesses two suckers in each extremity, a large adhesive posterior sucker and a small anterior sucker. It lacks a hard exo-skeleton and possesses thin flexible cuticle. So, it dries out quickly and that is why it is highly associated with water. However, there are also some species that are terrestrial, or land varieties. These are usually found on the surface of trees and grasses and under stones in damp places (1). Leeches occasionally enter the human orifices such as the eyes, nasopharynx, urethra, vagina and rarely the rectum, causing mucosal, orificial, vesical or internal hirudiniasis depending on the localization of the leech. Infestation usually occurs by drinking infested water or bathing in stagnant streams, pools or springs which are infested with leeches (1).

Leeches lead to anemia in two ways. The first one is by sucking of blood, a leech sucks out as much blood as ten times its own weight in 30 minutes. The second is by causing local bleeding which is due to pharmacologically active anticoagulant, hirudin and vasodilator substance, histamine, introduced by the leech bite. Hirudin is a polypeptide that inhibits the thrombincatalyzed conversion of fibrinogen to fibrin. Additionally, Hirudin blocks platelet aggregation in response to thrombin and may inhibit factor X (2). Leech in the rectum is a rare phenomenon, and so far, we found two case reports of pediatric and one adult patient with rectal leech infestation (3, 4). Hence, given the rarity of the case, we hereby report a case of a 4 years old male child presenting with lower gastrointestinal bleeding due to leech infestation.

## Case Report

This is a 4 years old male child who presented with painless rectal bleeding of two weeks duration. The bleeding was frank blood and not associated with defecation. He also had vomiting of ingested matter of three days duration. For this complaint, he visited a nearby health center after 4 days of onset of illness where he was given an unspecified tablet for three days, but there was no improvement. He had no bleeding from other sites and no previous similar episode. His father reported that he usually plays in a small river that the family uses for irrigation as well as drinking purposes. Cattle also drink from the same water source. Additionally, his father claimed that he had seen a leech around the child's rectum which he tried to remove manually but was not successful.

Physical examination revealed that he was underweight and tachycardic (pulse rate of 146 beats per minute). Additionally, he had severely pale conjunctivae, ejection systolic murmur at the apex and per rectal examination showed blood on examining finger. Laboratory investigations showed WBC = 34,320/µl (Neutrophil= 59.3%, Lymphocyte = 34.7), Heamatocrit = 9.2%, Platelet = 262,000/µl; Peripheral morphology - normocytic and normochromic RBCs, few macro-ovalocytes, and polychromatic spherocytes.

**Figure 1:**
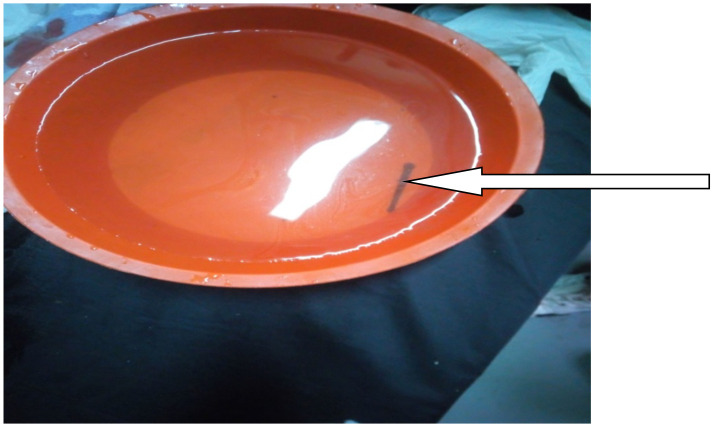
Leech removed from the rectum of a four years old child

With the diagnosis of severe anemia secondary to lower GI bleeding secondary to leech infestation (rectal), he was transfused with 20 ml/kg of whole blood and the child was asked to sit on a bucket of clean tap water during which the leech came out on its own into the water after around 15 minutes (Figure 1). The patient was observed for 24 hours, post-transfusion Heamatocrit was determined (19%) and he had no bleeding afterwards. Finally, he was discharged with therapeutic dose of iron sulphate and after parents were counseled on how to prevent similar episodes in the future.

## Discussion

Most leech attachments are short-lived, but leeches feeding on mucous membranes may stay in an orifice for days or weeks. Depending on the site of attachment, leeches may cause the host to exhibit haematuria, haemoptysis, haematemesis, hematochezia, epistaxis, dysphonia, coughing, tickling sensation and dyspnea. When the bleeding is prolonged and massive, it can lead to anemia (1). Leech in rectum presents with painless rectal bleeding, anorectal discomfort, crawling sensation in perianal area and tenesmus (3,4). Our patient had one of these symptoms, which is painless rectal bleeding for 2 weeks which probably led him to develop severe anemia.

Removal of leeches from the different orifices should be done carefully as the attempts to remove them might be associated with complications. For instance, removing leech with forceps is difficult because it has a soft and slippery skin, which ruptures easily. Additionally, firm traction should not be used when pulling a leech off because parts of its mouth may remain behind, leading to continuation of bleeding and secondary infection (5). In one of the case reports, leech hooklet from the rectum was removed by foreign body removal forceps after visualization with distal colonoscopy (3). In another report, removal was done by clamping the mouth with forceps and waiting for four minutes (4). In our case, the leech was removed by letting the child sit on a bucket of clean water for around 15 minutes after which the leech came out without any further manipulation. The possible mechanism behind this technique is that as some of the species of leeches are aquatic, when coming in close contact with the water surface, the leech might attempt to go to the water surface where it ultimately resides. Visualization of the leech and the possible site of attachment with proctoscope or colonscope which might have assisted in removal of the leech would have been helpful but was not done due to the unavailability of this equipment in the setting. Though rare, rectal leech infestation should be considered as one of the differential diagnosis of a child presenting with lower gastrointestinal bleeding.

## References

[1] YM (2013). Other Ectoparasites: Leeches, Myiasis and Sand Fleas. Manson's Tropical Diseases.

[2] Joslin J, Biondich A, Walker K, Zanghi N (2017). A Comprehensive Review of Hirudiniasis: From Historic Uses of Leeches to Modern Treatments of Their Bites. Wilderness Environ Med.

[3] Narayan J, Nath P, Singh A, Padhi PK, Parida PK, Pati GK (2017). Leech infestation presenting as severe rectal bleeding. J Dig Endosc.

[4] Behçet AL, Yenen Mehmet Emin, Aldemir Mustafa (2011). Rectal bleeding due to leech bite. Turkish Journal of Trauma & Emergency Surgery.

[5] Saki N, Rahim F, Nikaghlagh S, Saki G (2009). Meta Analysis of the Leech as a Live Foreign Body: Detection, Precaution and Treatment. Pakistan Journal of Biological Sciences.

